# Tailoring the structural, morphological, optical and dielectric properties of lead iodide through Nd^3+^ doping

**DOI:** 10.1038/s41598-017-16086-x

**Published:** 2017-11-23

**Authors:** Mohd. Shkir, S. AlFaify

**Affiliations:** 1 Advanced Functional Materials and Optoelectronic Laboratory (AFMOL), Department of Physics, College of Science, King Khalid University, Abha, 61413, P.O. Box 9004 Saudi Arabia; 2 Research Center for Advanced Materials Science (RCAMS), King Khalid University, Abha, 61413 P.O. Box 9004 Saudi Arabia

## Abstract

Hexagonal single crystal nanosheets of Nd^3+^ doped PbI_2_ were effortlessly synthesized via microwave-assisted technique under a power of 700 W and in a duration of 15 minutes with a homogeneous morphology. X-ray diffraction, energy dispersive X-ray spectroscope, scanning electron microscope, FT-Raman, UV-Visible, photoluminescence and dielectric measurement were employed to study the product. High purity, single phase and presence of Nd^3+^ doping was confirmed. SEM study confirm the formation of nanorods and single crystal nanosheets of very few nanometers in size. Robust vibrational analysis has been carried out and the observed bands are assigned to the vibration modes of E_2_^1^, A_1_^1^, A_1_^2^, 2E_2_^1^ and 2E_1_^1^, respectively. These bands are red-shifted when compare to the corresponding bulk values which indicate relaxed nanostructure formation and occurrence of confinement effect. The thickness of the synthesized single crystal nanosheets are found to be in the range of ~20 to 30 nm. The energy band gap was calculated and found to be 3.35, 3.34, 3.42 and 3.39 eV for pure, 1, 3 and 5% Nd^3+^ doped lead iodide, respectively. The clear blue luminescence has been observed at 440 nm and 466 nm when excited at 250 nm and 280 nm respectively. Dielectric and ac electrical conductivity was also measured and discussed.

## Introduction

In recent past, nanostructured semiconductor materials receives the colossal attention from scientists and researchers around the world due their wide range of applications in the field of environmental and energy applications, gas-sensing, field-emission, radiation detection, solar cell, and optoelectronic devices^1–5^. In particular, Lead iodide (PbI_2_) is a large band gap (2.3 eV) p-type semiconductor material and possess wide range of applications such as in active matrix flat panel imagers, room temperature radiation detection, especially for the low energy X-ray spectrometer, mammography energy range detection and nuclear particle detectors, photoconductors, photo-detectors, photovoltaic, co-precipitation sensors, biological labeling and diagnostic, light emitting diodes etc.^6,7^. In past decade, various research and development (R&D) activities were carried out on one-dimensional nanostructured materials based on varying forms including nanocrystals, nanorods, nanobelts, nanoribbons, nanowires, nanotubes etc. due their novel properties and impending applications in nano devices^8,9^. However, only few reports are available on the synthesis of PbI_2_ nanostructures including PbI_2_ nanorods or nanorods like particles by inverse micro-emulsion, microwave, hydrothermal and ultrasonic methods^3,5,10,11^, PbI_2_ nanocrystals by colloidal, hydrothermal and sol-gel route^12,13^, PbI_2_ nanoparticles by inverse micelles^14^, PbI_2_ single crystalline nanobelts by hydrothermal route^15^, and PbI_2_ quantum dots and clusters by chemical route^16–18^. Lately, the fabrications of nanosheets, in general, are commanding much attention from varying bodies of scientific communities and institutions because of their promising potentials in many advanced and futuristic technologies^19–21^. Hence, and by the same token, our research group has recently synthesized the Gd^3+^ and Cs doped PbI_2_ nanosheets by hydrothermal and microwave routes^5,22,23^. Embarking on the same research direction, the microwave-assisted rapid synthesis of Neodymium (Nd^3+^) doped PbI_2_ single crystal nanosheets with uniform morphology is going to be addressed and discussed in this article. In fact, synthesizing nano/thin films of key semiconductor materials like ZnO, TiO_2_, SnO_2_ with Nd^3+^ doping has resulted in modified and interesting properties as reported elsewhere^24–27^. Therefore, it would be of interest to scientists and engineers alike to investigate the effect of Neodymium (Nd^3+^) doping on the important PbI_2_ semiconductor materials. Moreover, as per the authors’ knowledge on the available literature concerning PbI_2_, this would be the first report on the microwave-assisted rapid synthesis of Neodymium (Nd^3+^) doped PbI_2_ single crystal nanosheets with uniform morphology. It is well known that the microwave-assisted synthesis offers a method to resolve the problem in which microwave provides a rapid and volumetric heating at higher reaction rate and better selectivity and very short reaction time in comparison of conventional heating^20,28,29^. The synthesized single crystal nanosheets of Nd^3+^ doped PbI_2_ were characterized by X-ray diffraction (XRD), scanning electron microscope (SEM)/energy dispersive X-ray spectroscopy (EDXS), FT-Raman and UV-Visible spectroscopies, Photoluminescence (PL) and impedance spectroscopy measurements. The obtained results are discussed, and the conclusion was drawn as well.

## Experimental details

### Chemicals

The analytical grade materials of Lead acetate (Pb(C_2_H_3_O_2_)_2_), sodium iodide (NaI), Sodium dodecyl sulfate (SDS), Poly(vinyl alcohol) (PVA), Neodymium (III) nitrate were purchased from CDH, Alfa Aesar, and Sigma Aldrich and used for the titled materials synthesis without further purification.

### Microwave-assisted synthesis

**Figure 1:**
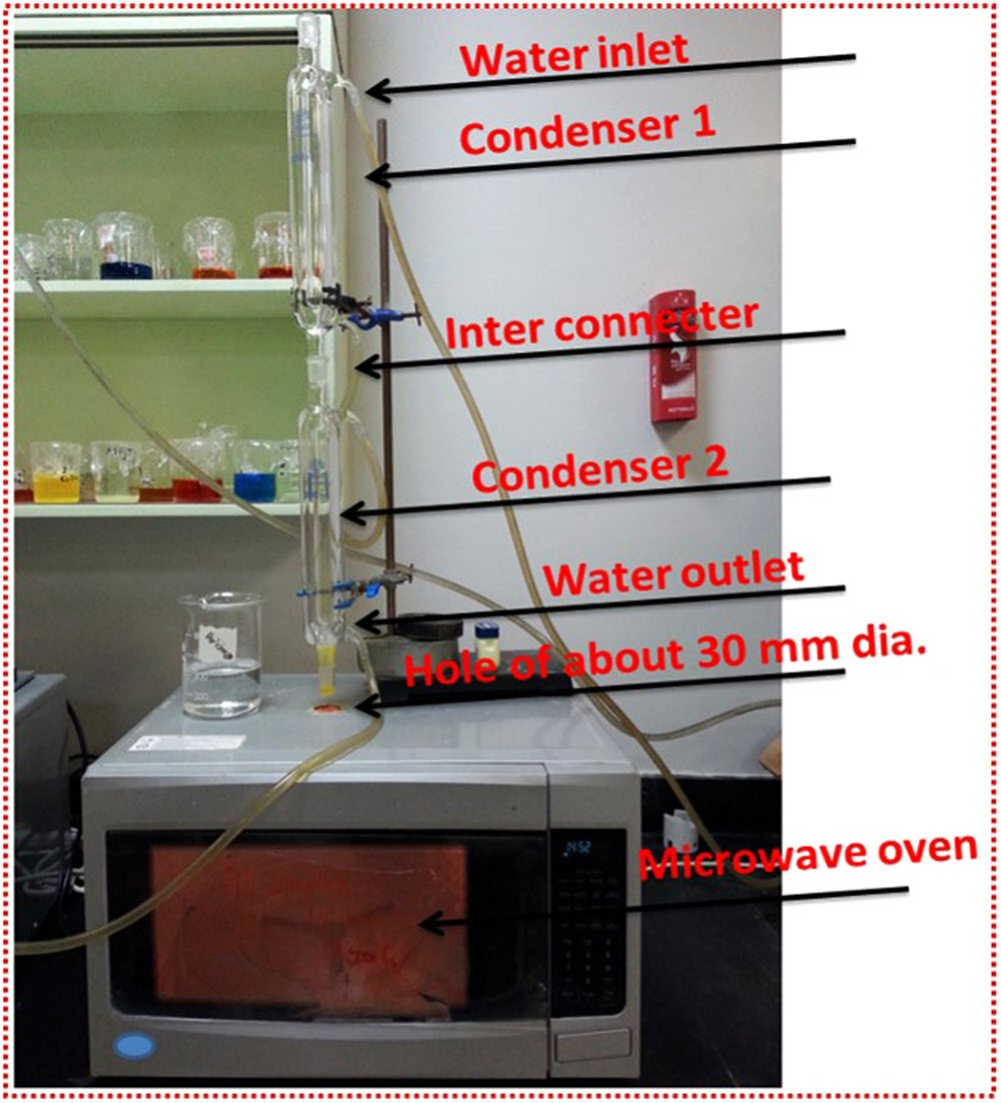
Used modified microwave system for nanosynthesis.

In a typical synthesis of pure and Nd^3+^ doped PbI_2_ nanorods and nanosheets the following steps were taken: (I) 0.5 M lead acetate (18.967 g) was dissolved in 50 ml double distilled water in a highly cleaned cylinder, once it was dissolved and clear solution appears, 50 ml SDS (10 g/ml) and 50 ml of PVA (45 g/liter) solutions were added one by one as surfactant and continuous stirring was performed at a stable magnetic stirrer fixed at 1000 rpm to get homogeneous solution at room temperature. The use of the surfactant is justified as it plays a key role in the synthesis of well-organized nanostructures and various kinds of surfactant have been used extensively in the past for the same purpose by many researchers and scientists^5,30,31^. (II) 1 M Sodium Iodide (14.988 g) was taken in another beaker and 50 ml distilled water used to dissolve it through stirring. Within 30 minutes, the homogeneous solutions are achieved, and finally, the prepared solutions were mixed together and stirred continuously. The temperature was set to 60 ± 2 °C during the whole synthesis process and within few seconds the solution becomes yellow which indicates the formation of PbI_2_. Preparing the other three solutions for doping purpose, the same latter procedure was used. (III) During the synthesis of PbI_2_, the different concentrations of Neodymium (III) nitrate [i.e. 1, 3 and 5 wt% of Nd^3+^ doping] were dissolved in 25 ml double distilled water in three different beakers and added to the PbI_2_ solutions at the same temperature. Finally, all the prepared solutions were reassign into cylindrical glass vessels for microwave- assisted synthesis. For microwave-assisted synthesis, a domestic microwave system of frequency 2.54 GHz bought from LG (Model No. MS5246VR/00) has been used, and the programming was done for 15 minutes at the fixed power of 700 W and afterword the microwave was automatically set off for natural cooling. The domestic microwave system was modified by making a hole at the center on the top of the furnace, and two condensers were used on the furnace and connect with the solution contained cylinder as shown in Fig. 1. The washing of the finally prepared yellow color salts with double distilled water was done many times during the filtration processes, and the obtained powder was dried in an oven at 100 °C for 24 h.

## Results and Discussion

### Morphological analysis

**Figure 2:**
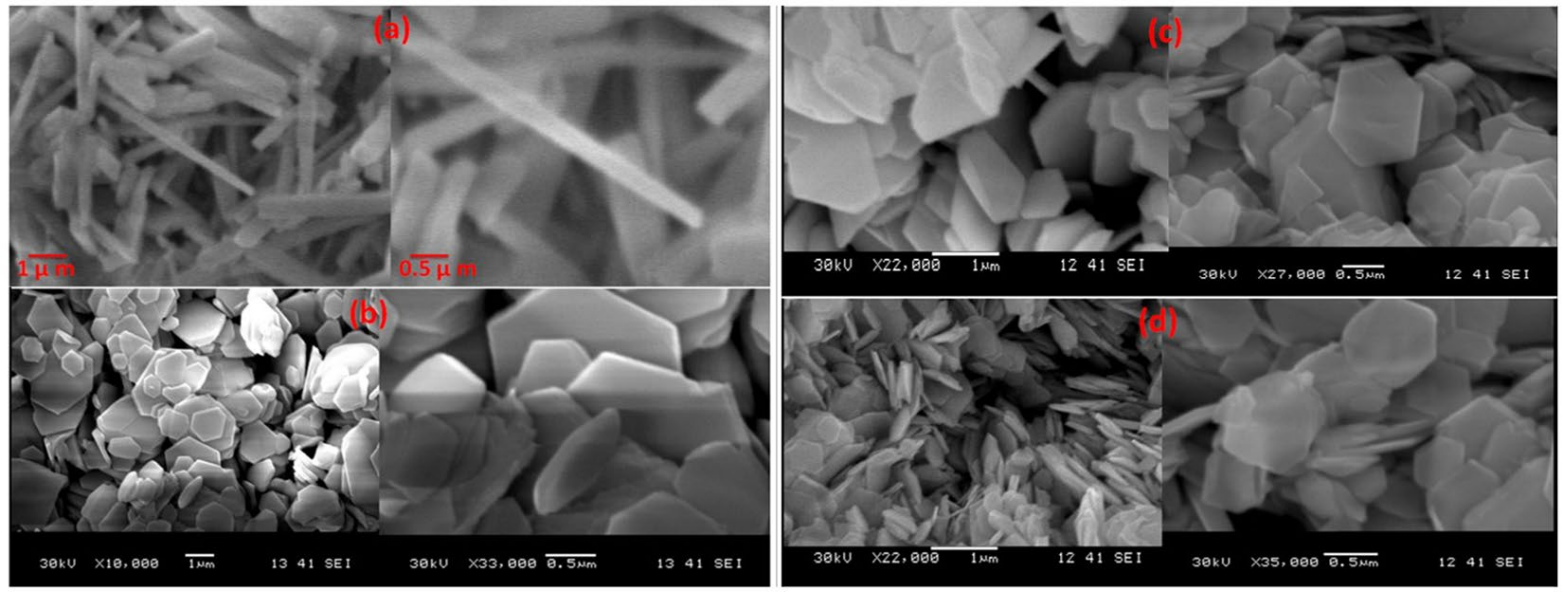
SEM micrographs of (**a**) pure and (**b**) 1 wt%, (**c**) 3 wt% and (**d**) 5 wt% Nd doped PbI_2_ nanorods and single crystals nanosheets.

Figure 2(a–d) shows the collected SEM micrographs of the pure and 1, 3, and 5wt.% Nd^3+^ doped PbI_2_ nanorods and nanosheets, respectively, captured at different resolutions/scales. As it is clearly visible from the Fig. 2(a), the morphology of the pure PbI_2_ is nanorods of diameters in the range of ~70 to 100 nm and length of few microns. However, the morphology of PbI_2_ has been remarkably changed from nanorods (as the case of the pure PbI_2_) to single crystal nanosheets of hexagonal shape (as it is the case of the Nd^3+^ doped PbI_2_) of thickness in the range of ~20 to 30 nm and width of about few nano- as well as micro-meters shown in Fig. 2(b,c and d). Apparently, Nd ions are playing the role of the heterogeneous seeds for the growing of the nanosheets. From SEM micrographs it is revealed that the synthesized nanostructures are of well-defined nanorods for pure PbI_2_ and single crystals nanosheets for Nd^3+^ doped PbI_2_ of homogeneous morphology. The scanning electron microscopy results confirms the strong effect of Nd^3+^ doping on the morphology of PbI_2_ nanostructures. However no major change in morphology or shape i.e. hexagonal structure, at higher concentration of Nd^3+^ doping has been observed, which means that by varying Nd^3+^ doping content within PbI_2_ the the single crystal nanosheets with the same hexagonal morphology the PbI_2_ material can still be achieved. The synthesized Nd^3+^ doped PbI_2_ nanosheets may provide the exceptional optoelectronic properties needed in many modern devices and applications^21^.

### X-ray diffraction analysis

**Figure 3:**
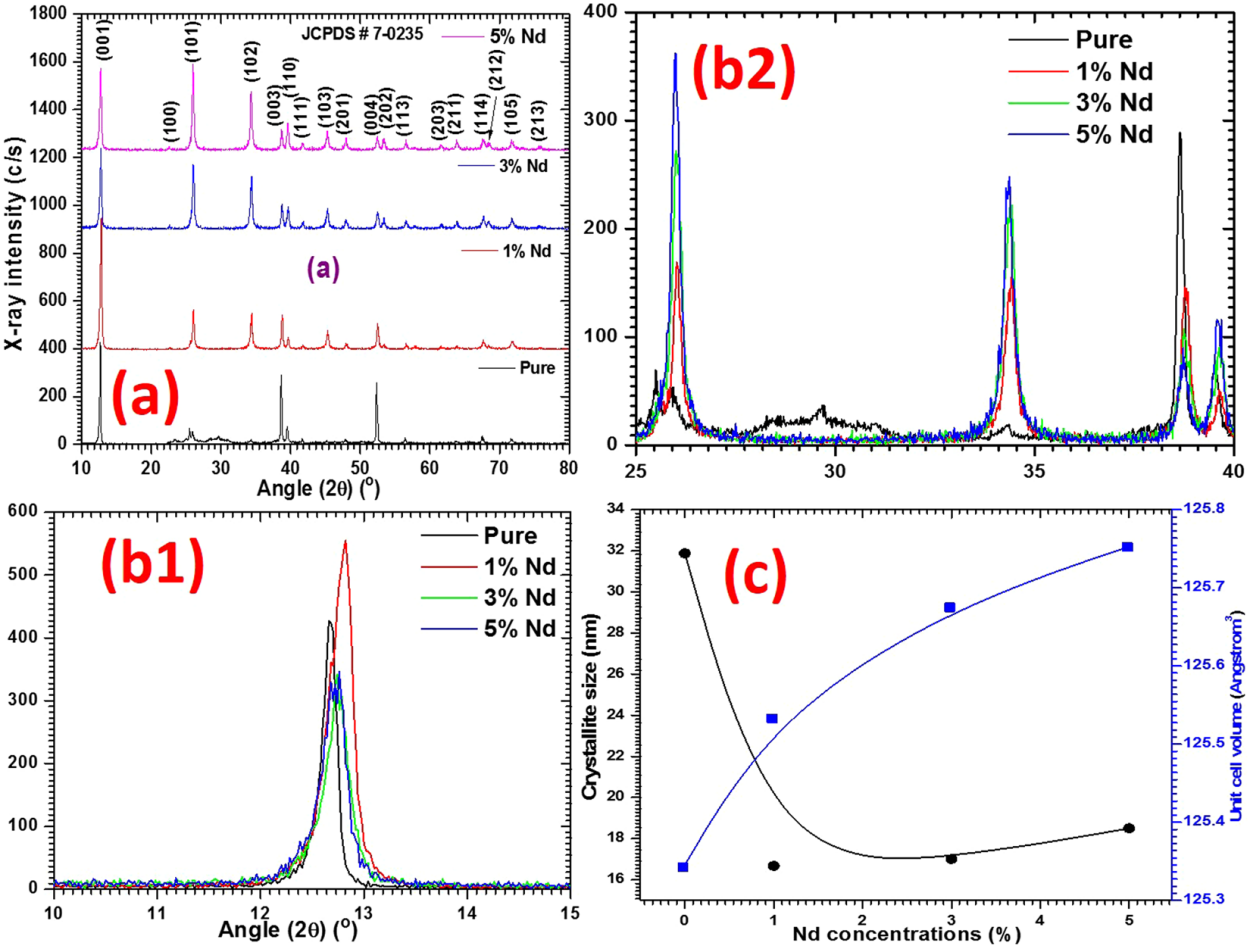
(**a**) X-ray diffractions patterns, (**b1** & **b2**) presentation of shifting in diffraction peaks and (**c**) Dependence of crystallite size of and unit cell volume as a function of Nd^3+^ doping.

The phase structure of the synthesized product (i.e. pure and 1, 3, 5 wt% Nd^3+^ doped) was examined by powder X-ray diffraction study. The recorded X-ray patterns are depicted in Fig. 3. The sharpness of the diffraction peaks proved good crystallinity within the fabricated nanostructures. The close inspection of X-ray diffraction pattern of the synthesized nanostructures confirms the high purity of the product as there is no peak due to other impurities like: PbO, PbOHI and Pb(OH)_2_ was observed. This was further confirmed by EDX/SEM mapping as well as FT-Raman studies presented in the forthcoming sections.

To understand the effect of Nd^3+^ doping on the crystalline structure of the PbI_2_, we have compared the X-ray diffraction patterns of pure and doped nanostructures as shown in Fig. 3(b1 & b2). The figure clearly indicates that the increase in Nd/Pb atomic ratio due to doping is having major influence on the preferential growth directions, specially along (101), of the nanostructures. Moreover, the relative peaks positions are slightly shifted in the case of doped PbI_2_ compared to the pure material. The intensity of peaks (101), (102), (103) and many others is found to be noticeably enhanced with doping. However, it can be noticed that there is a noticeable reduction in the peaks intensities, specially, for the (003) and (004) planes, and no additional peak due to impurity was observed, which confirms the absence of any free Neodymium nitrate in the prepared nanostructures. Furthermore, comparing the XRD patterns within Fig. 3(b1 & b2), a slight shift in the peak positions is observed in the doped nanostructures. This shift in the peaks position provides a clear indication of the change taking place within the lattice parameters of the grown nanostructure and lattice size due to Nd doping effect. The variation in lattice size of PbI_2_ suggests that Nd^3+^ ions can be incorporated into the crystalline matrix of the PbI_2_ structure either in substitutional or interstitial positions. To find out the nature of Nd^3+^ doping in the PbI_2_ lattice, we have calculated the lattice parameters (a, c) and grain size as a function of Nd^3+^ doping concentrations in PbI_2_ are presented in Table 1; the result is in close agreement with the standard literature values on PbI_2_ [JCPDS #7-0235]. The possible error in the refined parameters is totally based on the experimental procedure and environmental conditions and it may be around ± 0.001 Å. However, in the current work we have taken care about the condition to perform the X-ray measurements and also the refinement has been carried out many times. The nanostructures prepared by microwave-assisted route possess hexagonal phase of 2H-polytype of PbI_2_ with space group p-3m1 (164). The crystallite/grain size of the fabricated nanorods and nanosheets was calculated using Scherer’s formula: $D=\frac{k\lambda }{\beta \,\cos \,\theta }$, where, *k* = 0.9 and D is an average crystallite size, λ is X-ray wavelength (0.15406 nm), and β is full width at half maximum in radian. Furthermore, the values of lattice strain (ε) and dislocation density (**δ)** were also calculated from the following relations: ${\rm{\varepsilon }}\,=\frac{{\rm{\beta }}\times \,\cos \,{\rm{\theta }}}{4}$, and ${\rm{\delta }}=\frac{15{\rm{\varepsilon }}\,}{{\rm{a}}\times {\rm{D}}}$, respectively. The calculated values of D, ε and **δ** are given in Table 1. The variation of grain size and unit cell volume is shown in Fig. 3(c), from which it can be concluded that the unit cell volume is found to be increasing with increasing Nd^3+^ concentrations and hence lattice parameters a and c. Such variation can be explained with help of Vegard’s law, which suggests that when the incorporation process takes place substitutionally or interstitially within a crystal its lattice parameters are going to be shrinking or expanding, respectively^32^. In the present case, knowing the ionic radii of Nd^3+^ is 0.11 nm and Pb^2+^ is 0.133 nm, the lattice parameters of Pbi_2_ are found to be expanding due to doping which suggests the incorporation of Nd^3+^ ions within the interstitial sites of the crystalline matrix of the PbI_2_. We have not observed any amorphization even at higher concentration doping of Nd^3+^, which means that no disorientation was occurred in the prepared PbI_2_ nanostructures due to doping.

**Table 1:** Lattice parameters of pure and Nd^3+^ doped PbI_2_ nanorods and nanosheets.

Samples	a = b (Å)	c (Å)	V(Å)^3^	D (nm)	ɛ × 10^−4^ lin^−2^ m^−4^	δ × 10^15^ lines m^−2^
JCPDS- (07-0235)	4.557	6.979	125.511	—	—	—
pure_PbI_2_	4.554	6.978	125.342	31.871	0.110	0.0114
1 wt% Nd_PbI_2_	4.557	6.981	125.533	16.679	0.208	0.0410
3 wt% Nd_PbI_2_	4.559	6.983	125.675	17.008	0.204	0.0394
5 wt% Nd_PbI_2_	4.561	6.983	125.752	18.509	0.187	0.0333

### EDX and vibrational analyses

**Figure 4:**
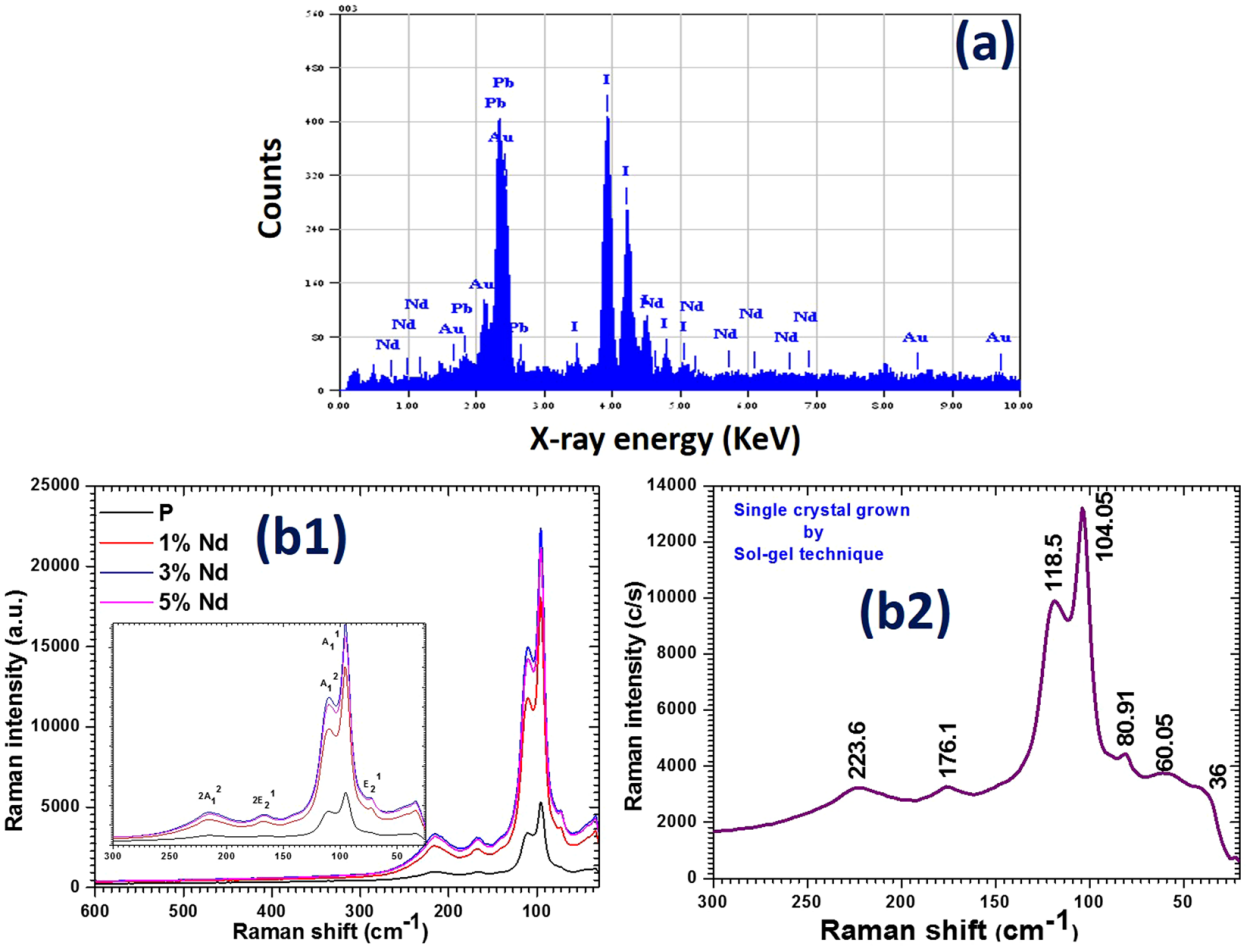
EDXS spectra for doped (**a**) and Raman spectra for pure and doped nanostructures (**b1**) and for single crystal (**b2**).

Figure 4(a) shows the recorded energy dispersive X-ray spectroscopy (EDXS) spectra for Nd^3+^ doped PbI_2_ nanosheets. As it is clear from figure that the peaks of Nd^3+^ are present in all the doped products. There is an extra energy peak due to Au in the pattern which was sputter on the samples for this measurement. Moreover, the SEM mapping was also carried out (see Fig. 1S supplementary data) to learn about the distribution of dopant inside the parent matrix and shows that the Nd doping has been taken places homogeneously in PbI_2_. Figure 4(b1) shows the recorded FT-Raman spectrum of the microwave-assisted synthesized pure (nanorods) and Nd^3+^ doped PbI_2_ nanosheets. From the figure, it is clear that no vibrational frequency is present in the Raman spectrum due to any impurity, which again proves that the grown material is high-quality nanostructure. The Raman peaks intensity is found to be enhanced in the case of the nanosheets structures at 1 wt% and 3 wt% Nd^3+^ doping, however at 5 wt% Nd^3+^ doping it was found to be reduced, but it is still higher than that of the pure PbI_2_. Such high intensity in Raman peaks indicates that the synthesized single crystal nanosheets are more crystalline than that of the nanorods structure of PbI_2_. The Raman bands for pure PbI_2_ (nanorods) are observed at ~73.13, 96, 111, 164, 213 cm^−1^ and for doped PbI_2_ (nanosheets) of 1 wt% Nd^3+^ at 73, 96, 110, 167, 216 cm^−1^, for 3 wt% Nd^3+^ at 73, 96, 110, 168, 215 cm^−1^, and for 5 wt% Nd^3+^ at 73, 96, 110, 168, 215 cm^−1^, and are assigned to vibration modes: E_2_^1^, A_1_^1^, A_1_^2^, 2E_2_^1^ and 2E_1_^1^, respectively. The observed bands confirms the formation of 2H-PbI_2_ Polytypes nanorods as well as single crystal nanosheets. These bands are found to be red shifted towards the lower wavenumber as compared to their corresponding values of the bulk material; they are also comparable with other reports on PbI_2_ nanostructures^33,34^. Furthermore, we have also grown the single crystal of PbI_2_ by gel method and recorded the Raman spectrum as shown in Fig. 4(b2). It can be clearly seen that the vibration bands in the grown single crystal are observed at 36, 60.05, 80.91, 104.05, 118.5, 176.1 and 223.6 cm^−1^, which shows a clear shifting in the position of Raman bands compared to the fabricated nanostructures of PbI_2_. The red shifting and broadening in the Raman bands of the synthesized both nanorods and single crystal nanosheets of PbI_2_ confirms the relaxed behavior of the grown nanostructure. Such red shifting and broadening can be explained in terms of the relaxation of the phonon wave vector q- vector, related to the lattice of the studied crystal within the framed theory of the Raman scattering in solid material. When the fundamental wave vector q ≈ 0, Raman selection rule is relaxed for a predetermined size domain or nanostructure. Such relaxation initiates contribution of phonons away from the Brillouin zone center of the crystal, according to the Heisenberg uncertainty principle. The uncertainty in phonon wave vector goes around as Δq ≈ 1/D, where D indicates the diameter or thickness of the synthesized nanostructures. This spatial confinement of optical phonon modes produces the red shifting and asymmetric broadening in active Raman peaks^35,36^.

### Optical studies

#### UV-Visible spectroscopic analysis

**Figure 5:**
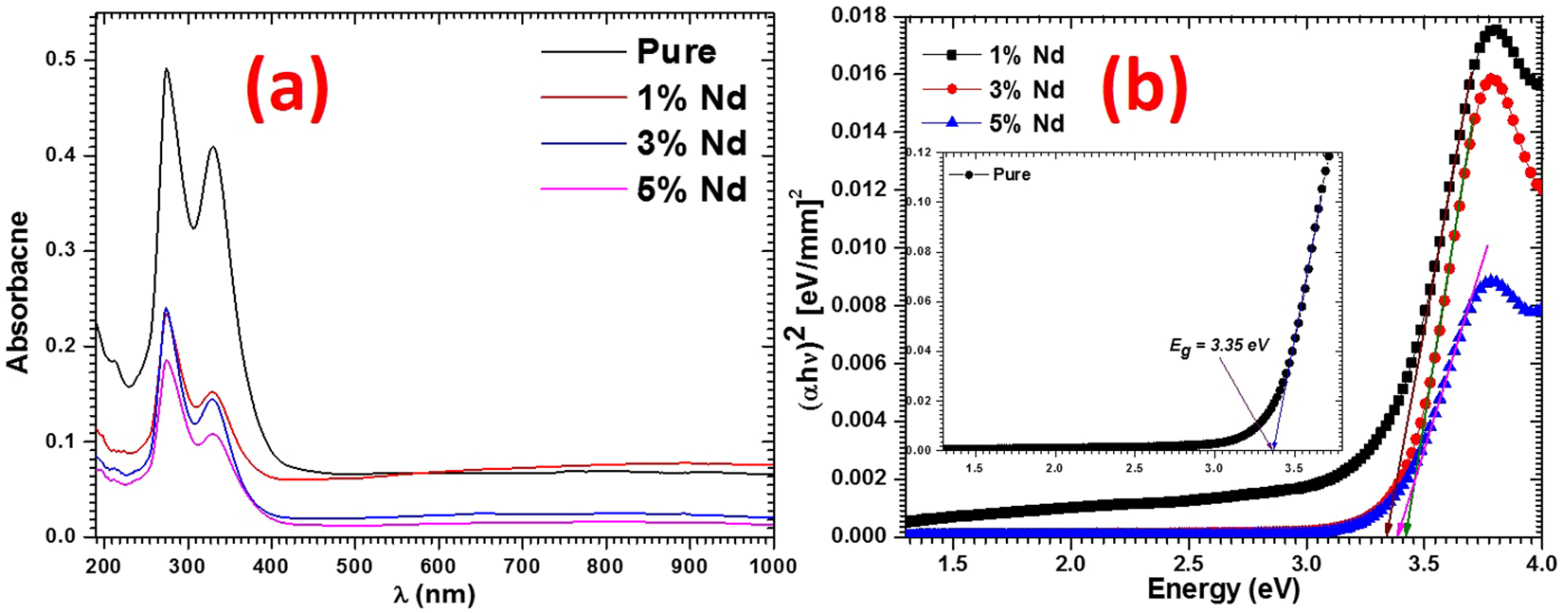
(**a**) Absorbance spectra and (**b**) energy band gap determination plots.

The absorbances of pure and doped PbI_2_ nanostructures were recorded by preparing their colloidal solutions using methanol as solvent as shown in Fig. 5(a). The energy band gap was evaluated from the recorded absorption spectrum in a transmission mode using the Tauc’s plot obtained from Tauc’s relation as shown in Fig. 5(b). To find out band gap from this plot we have followed the standard procedure, in which the value of the direct band gap E_g_, was determined to be correspond to the point of the intersection at (*αhv*)^2^ = 0, of the extrapolated straight portion of the curve (*αhv*)^2^ vs. *hv*, with the abscissa axis, where *hv* and *α* respectively measure the photon energy and absorption coefficient. For calculating *α* we have used well-known Beer–Lambert relation, *α* = 2.303*A/d*, where A is UV-visible absorbance and d is the path length of the quartz cuvette (10 mm). The optical band gap is found to be 3.35, 3.34, 3.42 and 3.39 eV for pure and for doped with 1 wt% Nd, 3 wt% Nd and 5 wt% Nd doped PbI_2_ nanostructures, respectively. The band gap value is not much effected by the 1 wt% Nd doping however, at 3 wt% and 5 wt% dopings it was found to be enhanced. It is clear that the values of band gaps of the PbI_2_ nanostructures are enhanced compared to that of bulk PbI_2_ value i.e. 2.3/2.27 eV^37^. The value of band gap for the fabricated nanostructures are enriched for about 1.07 ± 0.08 eV compared to bulk. This give a strong indication about the confinement effect taking place within the prepared nanostructures^38,39^. From such observation, we can assume that the UV-Visible method for extracting the optical properties is a suitable and more reliable technique for studying semiconductor nanostructured materials, particularly PbI_2_ since is not changing color even when doped; PbI_2_ has deep yellow color. Hence we will not get proper results on nanostructured PbI_2_ by the DR analysis, which provides the data from the materials surface only. In a previous studies on doped PbI_2_ in which we have reported band gap results using DR technique, one can see the discrepancies between DR values of the band gaps and the present results obtained by UV-Vis data^5,22^. The high value of the band gap of the nanostructured Nd doped PbI_2_ is a positive characteristic that will allow such material to be utilized in optoelectronic devices and it suggests that Nd doped PbI_2_ may be operated at higher voltages, frequency and temperature when compared to usual semiconductor materials. Due to such advantage of the fabricated nanorods and nanosheets of pure and Nd^3+^ doped PbI_2_, it may be used to fabricate a more powerful, cheaper and energy efficient electrical system^28,40–43^.

#### Photoluminescence (PL) analysis

**Figure 6:**
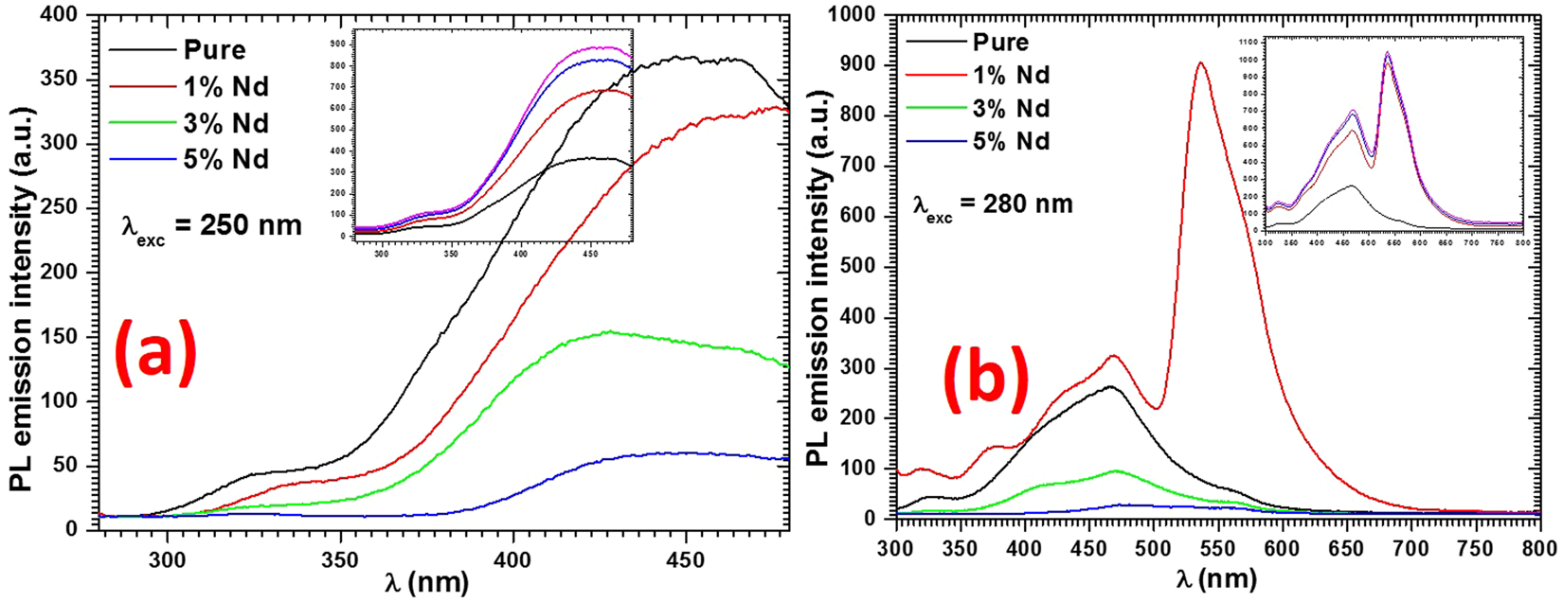
PL spectra for pure and doped PbI_2_ nanostructures.

Figure 6(a) and (b) shows the measured PL emission spectra for the fabricated nanorods and single crystal nanosheets of PbI_2_ at two excitation wavelengths, i.e., 250 and 280 nm. It may be mentioned here that the figures presented in the inset of figure (a) and (b) are for more clarity (cumulative) to get the right and more clear information. For PL measurement, the colloidal solutions of the fabricated nanostructures were prepared in methanol. It is evident from both figures that the emission spectra of pure and Nd doped PbI_2_ consists two emission peaks. The emission spectra recorded at 250 nm excitation wavelength have two broad peaks i.e. at ~325 ± 2 and 440 ± 2 nm in pure and Nd^3+^ doped PbI_2_. However, when such materials excited at 280 nm, there are three peaks which are at 325 ± 2, 466 ± 2 and 536 ± 2 nm in pure and Nd^3+^ doped PbI_2_. In this PL spectra some more bands are also observed as shoulder peaks at 409 nm and 563 nm in pure, while in Nd^3+^ doped nanostructures some more bands are also found at 373 ± 1 nm, 409 ± 2 nm and 577 ± 2 nm as shoulders with high intensity compare to pure one. Some of them confirm the presence of Nd^3+^ doping in PbI_2_, However, more details regarding this dopant in the titled materials can be observed in higher wavelength range as seen for the same doping in other materials^27^. In fact, more bands can be found for Nd^3+^, but that depends on the ability of detection of the instrument. The band observed at 325 nm is due to band edge emission and band observed at 409 nm is corresponding to energy emitted due to free excitons, E_F_, while other due to bound excitons, E_B_^38,44,45^. It can be noticed that when the fabricated nanostructures are excited at 250 nm, a blue luminescence at ~440 nm was observed. However, when excited at 280 nm this band is observed at ~466 nm in all of the prepared nanostructures. The PL intensity for 1% Nd^3+^ doped PbI_2_ is enriched remarkably, however for doped PbI_2_ at higher concentrations: the intensity of the PL spectra quenched down when excited at 280 nm. Such observations clearly indicates that there is a fundamental changes in the type and density of the defects within PbI_2_ due to doping, hence affecting the radiative and non-radiative recombination processes within the studied material due the increase in the increase of number of defects, dislocations, surface/interface and grain boundary states by the Nd^3+^ doping^27^.

### Dielectric and ac electrical conductivity analyses

**Figure 7:**
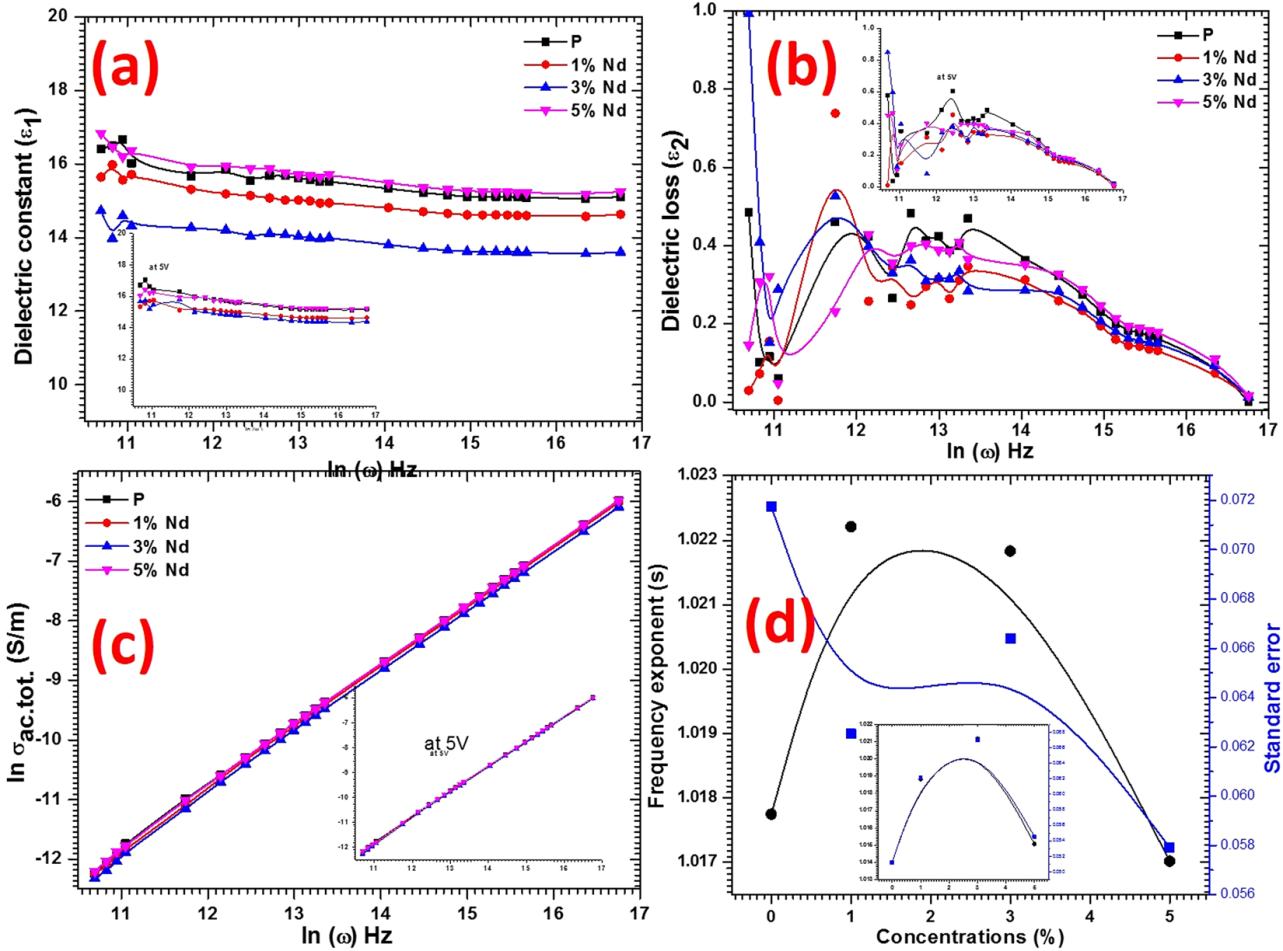
Plots for (**a**) *ε*_1_, (**b**) *ε*_2_, (**c**) ${\sigma }_{ac.tot.}$ and (**d**) s values of pure and doped PbI_2_ nanostructures.

For the dielectric functions and electrical conductivity studies, the capacitance (C), impedance (Z), and loss tangent (tanδ) are measured in the high-frequency range from 7 kHz to 3 MHz at 0 and 5 V bias voltage. Using the obtained data the dielectric constant (*ε*_1_) was calculated by the well-known formula: ${\varepsilon }_{1}=\frac{{\rm{C}}\times l}{{{\rm{\varepsilon }}}_{0}\times {\rm{A}}}$, where *l* is the thickness of the sample, ε_0_ is the vacuum space permittivity and A is the cross section area. Further, the dielectric loss (*ε*_2_) was evaluated using the relation: ${\varepsilon }_{2}=tan\,\delta \times {\varepsilon }_{1}$. The variation of the calculated values of *ε*_1_ and *ε*_2_ as a function of frequency at the 0 bs voltage are shown in Fig. 7(a) and (b), respectively. The dielectric functions calculated at 5 V bias voltage are also shown in the inset of Fig. 7(a) and (b). It is evident from Fig. 7(a) that *ε*_1_ values are almost constant in the entire testing frequency range for pure and Nd^3+^ doped PbI_2_ nanorods and single crystal nanosheets, which means that the electric field dipole does not follow the alternating field past a certain frequency for the studied material^46–50^. The high and stable value in the tested frequency range for the prepared nanostructures confirms their potential uses in optoelectronic devices applications. It can also be noticed from Fig. 7 that the values of *ε*_1_ are found to be lower, when compared to the *ε*_1_ values of the undoped PbI_2_, due to Nd^3+^ doping up to 3 wt%, however at 5 wt% Nd^3+^ doping the dielectric constants tended to be higher than that of the pure PbI_2_. The same type of trend was observed at the 5 V bias voltage. The average value of *ε*_1_ at 0 bias voltage for pure, and 1%, 3% and 5 wt% Nd^3+^ doped PbI_2_ are found to be ~15.5 ± 0.1, 14.5 ± 0.1, 13.5 ± 0.1 and 16.16 ± 0.1, respectively, however these values are found to be enhanced and higher than 14.5 ± 0.1 for all the grown nanostructures at 5 V bias voltage [see inset of Fig. 7(a)]. These values for the prepared nanostructures are found to be comparable as well as higher than the values reported for bulk PbI_2_^3,5,51^. The reduction in the value of *ε*_1_ at 1 and 3% Nd^3+^ doping is may be due to interfacial charge transfer. Such type of results were also reported previously for Nd^3+^ doped nanostructures^52^. Similar behavior was also observed for *ε*_2_ and can be seen in Fig. 7(b). The low loss values confirmed that the prepared nanostructures contain lesser defects. The alternating current total conductivity (${\sigma }_{ac.tot.}$) was determined from the impedance measurement by the following relation: ${\sigma }_{ac.tot.}=\frac{l}{Z\times A}$. Figure 7 (c) shows the variation of calculated ${\sigma }_{ac.tot.}$ with frequency. From figure it can be conclude that the value of ${\sigma }_{ac.tot.}$ is increasing with increasing the frequency, and following the universal power law of frequency. It is also found to have similar trends as *ε*_1_ and *ε*_2_ functions. Furthermore, the well-know Jonscher law, ${\sigma }_{ac.tot.}={\sigma }_{dc}+B{\omega }^{s}$ (where *σ*_dc_ is the direct current conductivity, B is a constant and *ω* is angular frequency and s is an exponent of frequency), was used to recognize the conduction behavior within the investigated material. The s values were noted for the grown nanostructures from the slope of linear part of the curve *ln* σ_*ac*_ *vs*. *lnω* [Fig. 7(c)] and presents in Fig. 7(d). The value of s is found to be increased first and then decreased with doping concentrations. It’s average value is about 1, which confirms that the hopping mechanism of conduction in the studied material involves a translational motion with sudden carrier hopping within the prepared material nanostructures.

## Conclusions

Facile and rapid synthesis of well-defined and homogeneous morphology nanorods and single crystal nanosheets of undoped and Nd-doped PbI_2_ have been achieved successful using microwave-assisted method for the first time. The X-ray diffraction and FT-Raman analysis confirms the formation of hexagonal phase of 2H-PbI_2_ polytypic. The presence of Nd^3+^ doping in the fabricated nanosheets of PbI_2_ was confirmed by EDX and EDX mapping analyses. SEM study confirms the formation of nanorods of diameter in the range of ~70 to 100 nm for the pure and single crystal nanosheets with a thickness in the range of ~20–30 nm for Nd^3+^ doped PbI_2_ with homogeneous morphology. A red shift in the vibrational modes of the Raman spectra of the grown nanostructures of PbI_2_ was observed in comparison to the respective modes of the bulk material, which clearly indicates the formation of the more relaxed nanostructure within the grown lead iodide structures. The values of the band gap (E_g_) calculated from UV-Visible data are found to be 3.35, 3.34, 3.42 and 3.39 eV for the pure and for the 1%, 3%, 5% - Nd^3+^doped PbI_2_ nanostructures, respectively. The PL spectra of the grown nanostructured PbI_2_ show a blue luminescence at ~440 nm when excited at 250 nm and at ~466 nm when excited at 280 nm in all fabricated samples. The average values of dielectric constant at 0 bias voltage are found to be ~15.5, 14.5, 13.5 and 16.16 for the pure, and for the 1%, 3% and 5 wt% Nd^3+^ doped PbI_2_ respectively. However, these values are found to be enhancing at 5 V bias voltage. The ac total electrical conductivity is also found to be enhancing with higher frequency. All obtained results suggest that the currently prepared single-crystal nanosheets of PbI_2_ are may be of great interest and potential for modern optoelectronic devices and applications.

## Electronic supplementary material

Figure 1S
