# Author Correction: Tailoring the structural, morphological, optical and dielectric properties of lead iodide through Nd^3+^ doping

**DOI:** 10.1038/s41598-018-28937-2

**Published:** 2018-07-11

**Authors:** Mohd. Shkir, S. AlFaify

**Affiliations:** 1 Advanced Functional Materials and Optoelectronic Laboratory (AFMOL), Department of Physics, College of Science, King Khalid University, Abha, 61413 P.O. Box 9004, Saudi Arabia; 2 Research Center for Advanced Materials Science (RCAMS), King Khalid University, Abha, 61413 P.O. Box 9004, Saudi Arabia

Correction to: *Scientific Reports* 10.1038/s41598-017-16086-x, published online 23 November 2017

The original version of this Article contained errors.

An additional affiliation was omitted for the authors Mohd. Shkir and S. AlFaify. The correct affiliations for these authors are listed below:

Advanced Functional Materials and Optoelectronic Laboratory (AFMOL), Department of Physics, College of Science, King Khalid University, Abha, 61413, P.O. Box 9004, Saudi Arabia

Research Center for Advanced Materials Science (RCAMS), King Khalid University, Abha, 61413, P.O. Box 9004, Saudi Arabia

Additionally, the Acknowledgements section was incorrect, where:

“The authors would like to express their gratitude to deanship of scientific research, King Khalid University, Saudi Arabia for providing administrative and technical support.”

now reads:

“The authors would like to express their gratitude to Research Center for Advanced Materials Science (RCAMS) - King Khalid University, Saudi Arabia for support.”

These errors have now been corrected in the HTML and PDF versions of the Article, and in the accompanying Supplementary Information file.

