# Supplementary material for: Tailoring the structural, morphological, optical and dielectric properties of lead iodide through Nd3+ doping
Source: Sci Rep. 2017 Nov 23;7:16091. doi: 10.1038/s41598-017-16086-x (PMC5700968; doi:10.1038/s41598-017-16086-x)

Tailoring the structural, morphological, optical and dielectric properties of lead iodide through Nd^{3+} doping

Mohd. Shkir*, S. AlFaify*

Advanced Functional Materials and Optoelectronic Laboratory (AFMOL), Department of Physics, College of Science, King Khalid University, Abha 61413, P.O. Box 9004, Saudi Arabia

Research Center for Advanced Materials Science (RCAMS), King Khalid University, Abha, 61413, P.O. Box 9004, Saudi Arabia

Figure 1S: SEM mapping image for Nd doped PbI_2 nanosheets

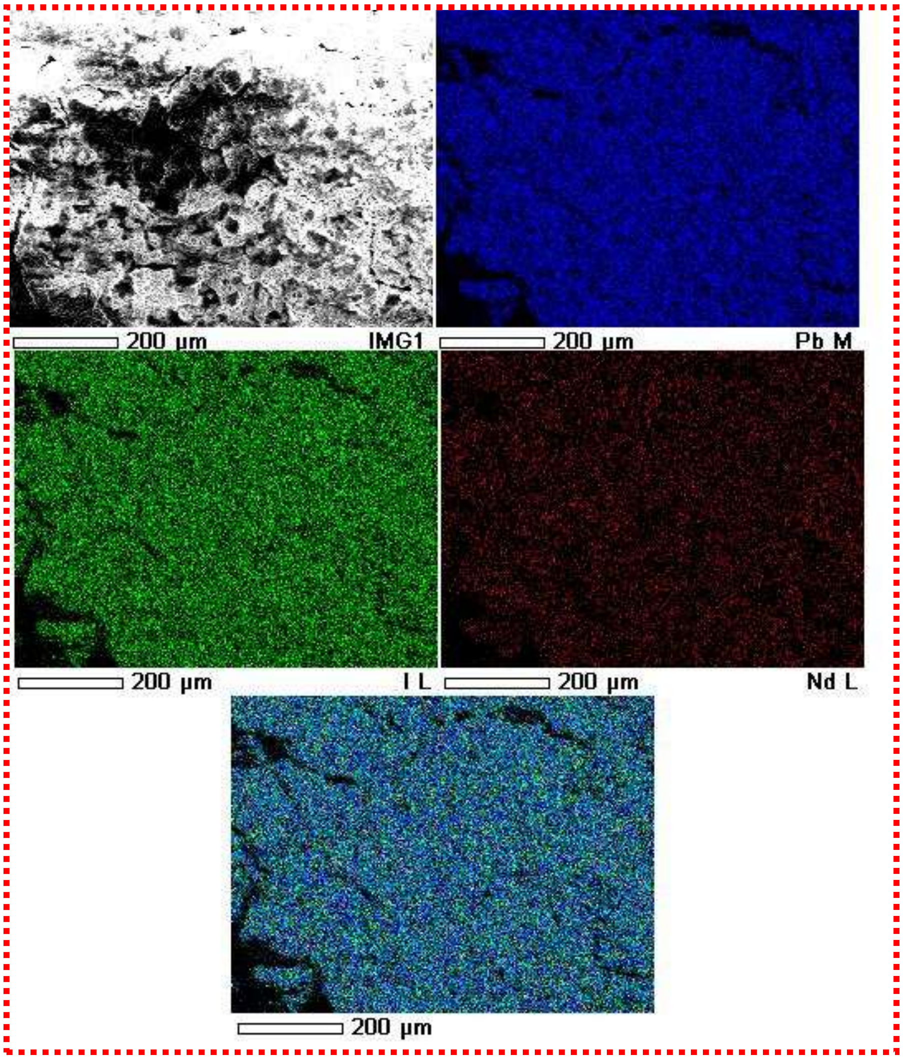

Supplement: Supplementary file 1 — Figure 1S [file 41598_2017_16086_MOESM1_ESM.pdf]
